# Elevated-CO_2_ Response of Stomata and Its Dependence on Environmental Factors

**DOI:** 10.3389/fpls.2016.00657

**Published:** 2016-05-13

**Authors:** Zhenzhu Xu, Yanling Jiang, Bingrui Jia, Guangsheng Zhou

**Affiliations:** ^1^State Key Laboratory of Vegetation and Environmental Change, Institute of Botany, Chinese Academy of SciencesBeijing, China; ^2^Chinese Academy of Meteorological SciencesBeijing, China

**Keywords:** elevated CO_2_, drought, guard cell, global warming, mesophyll-driven signals, regulation mechanism, photosynthesis, stomatal behavior

## Abstract

Stomata control the flow of gases between plants and the atmosphere. This review is centered on stomatal responses to elevated CO_2_ concentration and considers other key environmental factors and underlying mechanisms at multiple levels. First, an outline of general responses in stomatal conductance under elevated CO_2_ is presented. Second, stomatal density response, its development, and the trade-off with leaf growth under elevated CO_2_ conditions are depicted. Third, the molecular mechanism regulating guard cell movement at elevated CO_2_ is suggested. Finally, the interactive effects of elevated CO_2_ with other factors critical to stomatal behavior are reviewed. It may be useful to better understand how stomata respond to elevated CO_2_ levels while considering other key environmental factors and mechanisms, including molecular mechanism, biochemical processes, and ecophysiological regulation. This understanding may provide profound new insights into how plants cope with climate change.

## Introduction

Elevated atmospheric carbon dioxide concentration (elevated CO_2_) is a major component of climate change. It has increased from the pre-industrial level of 280 μmol mol^-1^ in 1750 to *c*. 400 μmol mol^-1^ at present and is expected to increase to *c.* 900 μmol mol^-1^ by the end of the 21st century. The global surface temperature is projected to rise 2.6–4.8°C by the end of this century, according to RCP8.5 (IPCC, 2013), a more undisciplined management scenario. Climate change, including elevated CO_2_, rising temperatures, and altered precipitation patterns, have markedly affected terrestrial ecosystem structure and function, carbon and water balance, and crop productivity (Lobell et al., 2011; Peñuelas et al., 2013; Ruiz-Vera et al., 2013; Bagley et al., 2015; Lavania et al., 2015). Moreover, a profound interaction between climate change and other critical environmental factors, including limited nutrition and air pollution, as well as some biotic factors, such as herbivorous insects, may intensify the adverse impacts (Gillespie et al., 2012; Peñuelas et al., 2013; Xu et al., 2013; Zavala et al., 2013; Sun et al., 2015; Xu et al., 2016).

Many studies have reported the biological responses to CO_2_ enrichment and their interaction with environmental change at various levels (Ainsworth and Rogers, 2007; Medeiros et al., 2015; Xu et al., 2015; Rodrigues et al., 2016). Elevated CO_2_ generally can enhance CO_2_ fixation and consequently plant growth and production (Ainsworth and Rogers, 2007; Xu et al., 2013). On the other hand, the decrease in stomatal conductance (*g*_s_) under elevated CO_2_ conditions may limit the CO_2_ fixation rate but promote water use efficiency (*WUE*) to benefit plant growth, especially within a climate change context where water shortage periods are expected to increase (Leakey et al., 2009; Sreeharsha et al., 2015).

Of these responses, the stomata are pivotal doors that control the gas exchange between vegetation and the atmosphere, i.e., CO_2_ entering from the atmosphere and water vapor releasing from plants into the atmosphere (Woodward, 1987). Carbon dioxide can reach the fixed Rubisco site through CO_2_ gas diffusions from the boundary layer, stomata, and intercellular airspaces near the chloroplast (Ball et al., 1987; Woodward, 1987; Warren, 2008). The main factors controlling stomatal opening processes include Ca^2+^ level, guard cell turgor, and hormones (Assmann, 1999; Lawson et al., 2014). Stomatal behavior may be affected by environmental factors, such as water status (e.g., soil water deficit, vapor pressure deficit [VPD]), temperature, CO_2_ concentrations, and light either alone and/or in combination (e.g., Lee et al., 2008; Perez-Martin et al., 2009; Hubbart et al., 2013; Laanemets et al., 2013; Šigut et al., 2015). Furthermore, stomatal short-term behavior (e.g., stomatal closure) and a long-term developmental (e.g., stomatal size and its density) responses to environmental changes might occur together, depending on species and genotypes (Gray et al., 2000; Ainsworth and Rogers, 2007; Haworth et al., 2013; DaMatta et al., 2016).

Our review focuses on the stomatal responses to elevated CO_2_ conditions with climatic change as well as the relevant metabolic processes and underlying mechanisms. The future perspectives for this study and possible implications are briefly presented and discussed. The present report may advance our current knowledge of the stomatal response to climatic change. It may also provide a new vision of its interdisciplinary and systematic synthesis to promote further relevant research.

## Stomatal Response to Elevated Co_2_

Elucidating the stomatal response to CO_2_ concentrations is important for understanding the stomatal physiology and gas exchanges between vegetation and the atmosphere. To adapt CO_2_ intake for photosynthesis and water release for transpiration, plants need to mediate stomatal development and behavior to balance CO_2_ and water exchange through the leaf epidermis in a changing environment (Gray et al., 2000; Haworth et al., 2013; Lawson et al., 2014). Elevated CO_2_ generally causes reductions in stomatal density (SD, e.g., Woodward, 1987; Lin et al., 2001; Teng et al., 2009), stomatal conductance (Medlyn et al., 2001; Ainsworth and Rogers, 2007; Gao et al., 2015), leaf transpiration (Teng et al., 2009; Katul et al., 2010), and canopy/ecosystem evapotranspiration (Medlyn et al., 2001; Bernacchi et al., 2007; Leakey et al., 2009; Bernacchi and VanLoocke, 2015). However, some studies have challenged this response because the reverse response might occur when elevated CO_2_ interacts with other climatic factors (see the sections below).

### Stomatal Conductance Response

#### Response Magnitude

The decreased magnitude of *g*_s_ by CO_2_ enrichment greatly depends on environmental variables and species (Medlyn et al., 2001; Ainsworth and Rogers, 2007; Haworth et al., 2013; Ward et al., 2013). In an earlier report, doubled ambient CO_2_ decreased *g*_s_ by *c.* 40% in almost all enclosure experiments, such as greenhouse and chamber experiments (Kimball et al., 1993; Morison and Lawlor, 1999). A 50% *g*_s_ decrease induced by elevated CO_2_ was found (Jackson et al., 1994), and a synthesis report showed a 21% *g*_s_ decrease in trees (Medlyn et al., 2001). A model scaling from leaf-level to canopy indicated that elevated CO_2_ might reduce canopy *g*_s_ by 16% (Baldocchi and Harley, 1995). According to a meta-analysis, the elevated CO_2_-induced *g*_s_ reduction in free air CO_2_ enrichment (FACE) experiments was averaged 22% across all plant species (*n* = *c*. 580). A significant variation among plant functional types (PFTs) was obtained: a maximum decease for C_3_ grass (30–40%) and a minimum decrease for shrub species (*c*. 15%; Ainsworth and Rogers, 2007). However, in a few experiments, *g*_s_ did not respond to CO_2_ concentrations in an obvious way (Ellsworth et al., 2011; Haworth et al., 2013; Ward et al., 2013; Bernacchi and VanLoocke, 2015; DaMatta et al., 2016). The *g*_s_ increase was even observed (Uddling et al., 2009) with short-term CO_2_ fertilization, for instance, in *A. thaliana* (Zinta et al., 2014). A recent experiment also found 23 and 18% *g*_s_ increases from elevated CO_2_ conditions in during vegetative and reproductive growth phases, respectively, of the Pigeon pea (*Cajanus cajan* L.; Sreeharsha et al., 2015). In a recent finding, the *Arabidopsis* Tetraploid, Me-0, with larger stomata, still had a comparatively high *g*_s_ when exposed to increased CO_2_ concentrations, suggesting that taller plants with larger stomatal size can better deal with rising CO_2_ by improving their stomatal behavior (Monda et al., 2016). Thus, the decrease in *g*_s_ due to elevated CO_2_ is a *general* rather than a *universal* response due to some unexpected factors’ effects. This difference is particularly found in dramatic ecotypes-, species-, PFTs-, and development stages. As such, the underlying mechanism remains to be clarified further.

#### Interaction of *g*_s_, *A*, and *WUE*

The decrease in *g*_s_ generally leads to a decrease in net assimilation rate (*A*) and is recognized as one of the two major limitations of photosynthesis; the other is non-stomatal limitation (Noormets et al., 2001). There was no obvious evidence from FACE that *g*_s_ independently acclimated to elevated CO_2_ levels despite exposure time (Nijs et al., 1997; Leakey et al., 2006a; Ainsworth and Rogers, 2007; Gao et al., 2015). An earlier model by Ball et al. (1987) predicted that *g*_s_ may be restricted when down-regulation in *A* occurs in response to CO_2_ enrichment. Although stomatal limitation to photosynthesis may decrease with elevated CO_2_ levels (e.g., Noormets et al., 2001), the uncoupling of *g*_s_ with *A* has been confirmed in a transgenic tobacco plant due to its reduced Rubisco content (von Caemmerer et al., 2004). However, an experiment has shown that a high *A* caused by increasing *g*_s_ can be maintained in a rice mutant that has a deficient slow anion channel 1 (SLAC1), that is, a guard cell anion channel protein that does not respond to rising CO_2_ levels (Kusumi et al., 2012). Furthermore, a recent experiment indicated that, with elevated CO_2_, *Cajanus cajan* leaves had 7–18% higher leaf instantaneous *WUE* (*WUE*_i_) due to simultaneously maintaining both higher *A* and *g*_s_. However, the former was higher than the latter (Sreeharsha et al., 2015). It is also noteworthy that at a high CO_2_ levels, a significant *g*_s_ decrease in C_4_ plants, such as maize, may occur only during drought, leading to *WUE* promotion rather than enhanced photosynthetic capacity as a result of the *g*_s_ decrease (Leakey et al., 2006b, 2009). A recent report by Lawson and Blatt (2014) has indicated that although stomatal responses to environmental changes may be closely associated with CO_2_ assimilation and water transpiration, a better balance between CO_2_ uptake and water loss may be improved by manipulating guard cell physical, anatomical, and transport characteristics to promote *WUE* (Lawson and Blatt, 2014). This may need further testing under elevated CO_2_ conditions.

### Stomatal Development and Its Density

#### Response Magnitude of Stomatal Density

A decrease in SD is considered a general response to elevated CO_2_. As reported by Woodward (1987), as CO_2_ levels from the pre-industrial level of 280 μmol mol^-1^ rose to the ambient level of 340 μmol mol^-1^ in 1987, a dramatic (67%) decrease in SD was found in the leaves of herbarium specimens and in experiments under controlled environmental conditions. Based on a paleobotanic analysis of fossil *Buxus* (3775–3640 BC) by Rivera et al. (2014), the SD and stomatal index (SI) had significantly greater values than the current *Buxus balearica* and *B. sempervirens* species (297.6 vs. 227.8 stomata mm^-2^, 12.7 vs. 8.0%, respectively). The dramatic declines are closely associated with a drastic increase in atmospheric CO_2_ concentration that has been occurring since the mid-Holocene era (Joos et al., 2004; Rivera et al., 2014). However, only a 5% SD decrease due to elevated CO_2_ was obtained from a meta-analysis on stomatal response (Ainsworth and Rogers, 2007). Relatively few studies reported an unchanged (Tricker et al., 2005) or even increased SD (Reid et al., 2003). A recent report by Field et al. (2015) showed that SD in non-vascular land plants, such as hornwort (*Anthoceros punctatus*, *Phaeoceros laevis*) and some moss sporophytes, did not respond to CO_2_ enrichment. It was even slightly increased in *Funaria hygrometrica* sporophytes at elevated CO_2_. A recent report showed the appearance of SD responses to elevated CO_2_ depends on tropic coffee genotypes (Rodrigues et al., 2016). These findings imply that the magnitude of SD response to CO_2_ enrichment might easily vary according to the experimental facility, experimental duration, species/genotypes, and other environmental variables (e.g., Ainsworth and Rogers, 2007; Haworth et al., 2013; Rodrigues et al., 2016). Thus, considerable caution is required when using SD as an indicator of a stomatal adaptive process in response to elevated atmospheric CO_2_ concentration.

#### Stomatal Development under Elevated CO_2_

The relevant genes may be involved in stomatal development under elevated CO_2_ conditions (Gray et al., 2000). The *Arabidopsis* gene *hic* (high carbon dioxide) encodes a negative regulator of stomatal development that responds to CO_2_ concentrations and can be adversely regulated by elevated CO_2_. A 42% increase in SD in the mutant *hic* plants was evidence of a doubled CO_2_ level (Gray et al., 2000). *Arabidopsis* plants with the *GTL1* gene have higher transpiration and lower *WUE* due to regulation of SD via transrepression of SD and distribution 1 (SDD1; Yoo et al., 2010). As reported by Engineer et al. (2014), the extracellular pro-peptide-encoding gene epidermal patterning factor 2 (EPF2) in wild-type *Arabidopsis* can be induced by elevated CO_2_, possibly providing an essential role for CO_2_ control of stomatal development. Furthermore, in the β-carbonic anhydrase double mutants (*ca1*, *ca4*), a secreted protease CRSP may cleave the EPF2 and then repress stomatal development, demonstrating an inverse response of the wild-type plants to elevated CO_2_. This partly elucidates the key mechanisms of how the sensing and transduction CO_2_ signals are linked to stomatal development (Engineer et al., 2014). This finding also indicates that some transduction signals between stomata and nearby pavement cells (PCs) may be involved in abscisic acid (ABA)-mediated inhibition of PC enlargement and may ultimately affect stomatal distribution and its density (Tanaka et al., 2013a). It has been suggested that the signals are peptide hormones (Hunt et al., 2010; Sugano et al., 2010; Jewaria et al., 2013; Lee et al., 2015; see below). However, a clear role of the response of stomatal development to elevated CO_2_ remains largely unknown. A description on the genes regarding stomatal development in response to CO_2_ concentration are listed in **Table 1.**

**Table 1:** Selected genes related to stomatal development and movement responses to elevated CO_2_.

Species	Gene name	The genes description and/or regulating	Responses to rising CO_2_ and/or notes	Reference
*Arabidopsis thaliana*	*HIC*	A negative regulator of GCs	↓Stomatal development; ↓SD	Gray et al., 2000
*Oryza sativa*	*EP3*	GCs development	↑Stomatal development; ↑SD	Yu et al., 2015
*A. thaliana*	*GTL1*	Transrepression of SDD1	Possible lower transpiration and higher *WUE* by regulating SD	Yoo et al., 2010
*A. thaliana*	*EPF2*	An extracellular pro-peptide-encoding gene	↓Stomatal development through CA1, CA4, and CRSP; ↓SD; tuning stomatal patterns	Doheny-Adams et al., 2012; Engineer et al., 2014; Lee et al., 2015
*A. thaliana*	*STOMAGEN*	A positive response to stomatal development	↑Stomatal development;↑SD	Hunt et al., 2010; Doheny-Adams et al., 2012; Tanaka et al., 2013a
*A. thaliana*	*OST1*	A positive regulator of CO_2_-induced stomatal closure; activation of SLAC1	↑Stomatal closure; activation of the S-type anion channels	Xue et al., 2011; Negi et al., 2014
*A. thaliana*	C*A1, CA4*	Stomatal development and SD decrease in mutant plants	↓Stomatal development and movements; ↑Stomatal closure by stimulating K^+^ outward channel	Hu et al., 2010; Negi et al., 2014
*A. thaliana*	*SCAP1*	Dof-type transcription factor (AtDof 5.8); involving stomatal functioning, and maturing	↓Stomatal development?; ↑Stomatal closure; ↑K^+^ efflux from GCs	Negi et al., 2013, 2014; Medeiros et al., 2015
*A. thaliana*	*HT1*	Protein kinase, an RHC1 MATE-type transporter	↑Stomatal closure; a critical regulator of stomatal CO_2_ signaling	Hashimoto et al., 2006; Negi et al., 2014; Tian et al., 2015
*A. thaliana*	*ARPC2*	ARPC2 subunit of the ARP2/3 complex	Mediating GCs actin; ↑Stomatal closure?	Jiang et al., 2012
*A. thaliana; O. sativa*	*SLAC1*	S-type anion channel in the transmembrane region providing or regulating a gate for anion transport	↑ABA- and Ca^2+^-induced stomatal closure; ↑K^+^ efflux from GCs	Negi et al., 2008, 2014; Vahisalu et al., 2008; Kusumi et al., 2012; Yamamoto et al., 2016
*A. thaliana*	*PATROL1*	A Munc13-like protein tethering H+-ATPase to the PM.	↑Stomatal closure; controlling H^+^-ATPase to make H^+^ into GCs; translocated to cytoplasm	Hashimoto-Sugimoto et al., 2013; Negi et al., 2014
*A. thaliana*	*AtALMT12/QUAC1*	A member of the aluminum-activated malate transporter; targeted PM ion channel	↑Stomatal closure; ↑ABA response	Meyer et al., 2010
*A. thaliana*	*AtABCB14*	A malate uptake transporter into GCs	↓Stomatal closure; decreasing malate level	Lee et al., 2003, 2008
*A. thaliana*; *Vicia faba*	ROP2	Negative regulator of stomatal responses	↓Stomatal closure-induced by high CO_2_; but ROP2 can be inactivated by ABA	Hwang et al., 2011


*GCs, guard cells; PM, plasma membrane; SDD1, stomatal density and distribution 1; SD, stomatal density; ?, a possible response; ↑ and ↓, either promoted or retarded responses.*

#### Stomatal Density and *A* under Elevated CO_2_

Photosynthetic capacity is closely linked to SD (Xu and Zhou, 2008). Leaf *A* was negatively correlated with SD when plants were exposed to elevated CO_2_ (Woodward, 1987; Ainsworth and Rogers, 2007), whereas a positive correlation occurred when grass was subjected to a water status gradient (Xu and Zhou, 2008). Moreover, photosynthetic potential might be enhanced with increased SD in *Arabidopsis* by a modulating gas diffusion function, as was recently reported by Tanaka et al. (2013b). In this case, the *A* increase at elevated CO_2_ is tightly associated with increased SD. Here, the *stomagen* gene overexpression confers a positive response to stomatal development in *A. thaliana* (Hunt et al., 2010; Doheny-Adams et al., 2012; Tanaka et al., 2013b). As recently reported, an *EP3* gene in rice may be responsible for guard cell development, which may determine SD. This is due to the *ep3* mutant plants exhibiting a smaller GC with low SD, *g*_s_, and *A* compared with their wild-type controls (Yu et al., 2015).

#### Trade-off between Stomatal Density and Leaf Growth under Elevated CO_2_

**FIGURE 1:**
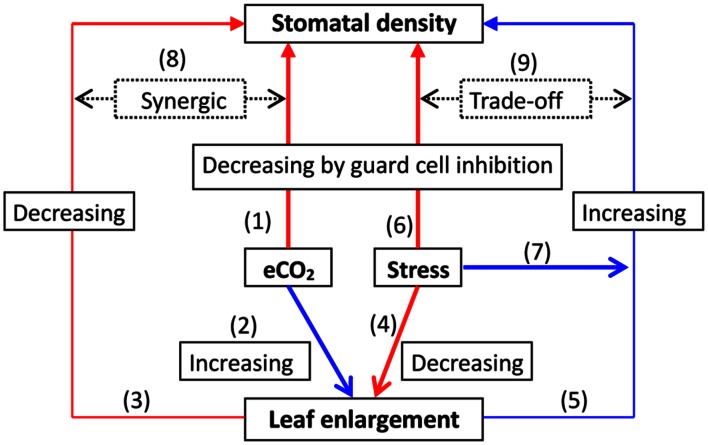
**A representation of the response to elevated CO_2_ (eCO_2_) with abiotic factor stress on stomatal density (SD) under regulation by leaf growth.** Elevated CO_2_ may lead to an acclimated reduction in SD by inhibition of guard cells, which involves the regulation of gene expression and adaptive evolution (1); meanwhile, eCO_2_ could promote leaf enlargement (2) consequently decreasing SD (3). A severe abiotic stress factor such as drought may diminish leaf enlargement (4), ultimately increasing SD (5); it may directly decrease SD due to inhibition of guard cell development (6). However, under moderate stress, SD may be increased possibly due to an acclimated response (7). In summary, this synergic increase (8) or trade-off interaction (9) may occur between the effects of both leaf growth and changes in SD toward the variations in eCO_2_ under abiotic stress, ultimately determining SD.

The response and feedback of SD with leaf growth to elevated CO_2_ may be described generally in a linkage network (**Figure 1**). A general SD decrease in CO_2_ enrichment may have several possible coherent explanations. (1) The promotion of a leaf area may contribute to a lower SD. For instance, the leaf area in grass plants markedly increased under elevated CO_2_ (Xu et al., 2014), possibly reducing the SD (Xu and Zhou, 2008; Xu et al., 2009b). An 11–23% decrease in SD by in Scots pine (*Pinus sylvestris*) needles by high CO_2_ conditions might result from an increase in needle thickness and needle width, i.e., the surface area of the entire needle. Thus, this structural plasticity may often occur in short-term elevated CO_2_ fertilization. (2) With long-term elevated CO_2_, the relevant gene expression levels may contribute to the diminishment of stomatal development, leading to a reduction in SD (e.g., Gray et al., 2000; Engineer et al., 2014). (3) The coordination of other key environmental factors may together regulate changes in SD. For instance, a moderate water deficit may increase SD due to a potential acclimated response, whereas excessive watering or severe water deficit stress decreases SD by inhibiting GCs (Xu and Zhou, 2008; Xu et al., 2009b). This suggests that the former would encounter an SD decline due to rising CO_2_, and the latter would accelerate its reduction further (Woodward, 1987; Xu and Zhou, 2008; Xu et al., 2009b).

Similarly, a temperature higher than optimum may limit leaf enlargement, leading to increased SD, but moderate warming may do the opposite. In fact, both temperature and water status or their interaction might regulate stomatal development and distribution in response to CO_2_ enrichment. This would ultimately determine SD due to the trade-off, depending on environmental stresses or species-specific adaptation (Fraser et al., 2009; Xu et al., 2009b; Locosselli and Ceccantini, 2013; **Figure 1**), which still remains elusive to some extent. As reported by Pyakurel and Wang (2014), elevated CO_2_ can reduce the leaf area and increase the SD of birch plants, demonstrating high resistance to water deficit stress. Moreover, an interaction between elevated CO_2_ and light may also determine SD. For example, rice leaf SD was slightly decreased by elevated CO_2_ or by decreased light irradiance. However, the effect of light on SD may be diminished by elevated CO_2_ (Hubbart et al., 2013). Thus, multifaceted effects on SD responses to elevated CO_2_ need to be further clarified.

### Molecular Mechanisms Controlling Guard Cell in Response to Elevated CO_2_

#### General Molecular Mechanism

**FIGURE 2:**
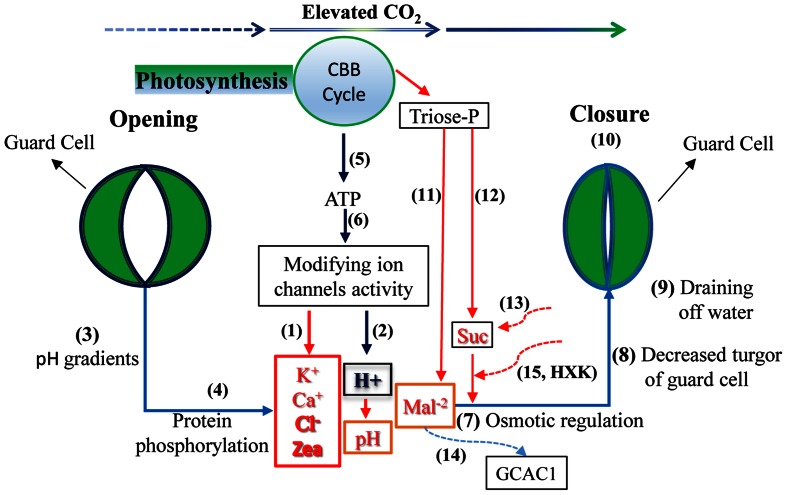
**Possible stomatal response mechanisms controlling guard cells (GC) under elevated CO_2_.** With rising CO_2_, a depolarization in GCs appears: the levels of K^+^, Ca^2+^, Cl^-^, and zeaxanthin (Zea) may decrease (1), whereas the H^+^ concentration may remain at a high level (2) leading to a lower pH value. The pH gradient (3), protein phosphorylation (4), and photosynthesis-derived ATP (5, 6) are involved in the regulation process by modifying channel activities; together, they promote osmotic regulation (7) and decrease GC turgor (8) consequently causing the GCs to drain water (9) leading to stomatal closure to some extent (10). Meanwhile, Calvin–Benson–Basshan (CBB) cycle and sugar metabolism in GC may produce less malate (Mal^2-^), (11) and sucrose (Suc) (12) with triose phosphate (triose-P) at eCO_2_, which also affects osmotic regulation. Furthermore, elevated CO_2_ may reduce Suc accumulation in the vicinity of the GC wall from the mesophyll due to the limitation of some apoplastic Suc in the transpiration stream toward GC (13) and enhance Mal^2-^ transport from GCs into mesophyll cells by stimulating anion efflux through channels such as GCAG1 and the potential involvement of the *AtABCB14* gene (14), also resulting in stomatal closure. Finally, hexokinase (HXK) involvement may limit sugar synthesis and its entrance into GCs from mesophyll cells (15) and then inducing stomatal closure (e.g., Webb et al., 1996; Assmann, 1999; Schroeder et al., 2001; Kang et al., 2007; Lee et al., 2008; Kim et al., 2010; Fujita et al., 2013; Kelly et al., 2013; Lawson et al., 2014; Negi et al., 2014).

Guard cell (GC) metabolism and the signal transduction network have been reviewed in several reports (e.g., Lawson et al., 2014; Negi et al., 2014). Here, we succinctly present the findings of these reports, particularly the explanations concerning the regulation of CO_2_ concentration (**Figure 2**). Generally, ion and organic solute concentration levels determine the turgor pressure of guard cells and subsequently affect stomatal aperture. Under elevated CO_2_, stomata tend to close because a greater depolarization seems to appear in GCs. The process may be controlled by (1) a decrease in K^+^ concentration, with enhanced activity in outward rectifying K^+^ channels and decreased inward activity, (2) decreased cytosolic Ca^2+^ in GCs, (3) decreased Cl^-^ and malate (Mal^2-^) concentrations by stimulating the release of Cl^-^ and Mal^2-^ from GCs resulting from the activation of S-type anion channels, and (4) by decreases in the cytosolic zeaxanthin level and the pH value in GCs. Together, these factors lead to a decline in GC turgor, causing the GCs to shrink and the stomatal aperture to close (e.g., Webb et al., 1996; Zhu et al., 1998; Assmann, 1999; Schroeder et al., 2001; Fujita et al., 2013; Lawson et al., 2014). The potential messengers in the stomatal response to CO_2_ concentrations mainly include ion channel activity, cytosolic free calcium, ABA, malate levels, membrane potential, pH gradients, zeaxanthin content in chloroplasts, photosynthesis-derived ATP content, protein phosphorylation, and dephosphorylation processes (McAinsh et al., 1990; Schroeder et al., 2001; Ainsworth and Rogers, 2007; Kim et al., 2010; Wang et al., 2013; Lawson et al., 2014). For instance, the experiments have shown that elevated CO_2_ can enhance anion channel activity in GCs to induce stomatal closure. In this event, the SLAC1 protein provides or regulates a gate for anion transport (Raschke et al., 2003; Marten et al., 2008; Vahisalu et al., 2008; Negi et al., 2014; Yamamoto et al., 2016).

#### Role of Sugar

In the guard cell itself, major reports have provided evidence that GCs may play only a trivial role in the regulation of the stomatal aperture, including osmotic adjustments. As such, GSs have a limited photosynthetic capacity, fewer chloroplasts, low electron transport, and relatively lower levels of relevant metabolites, such as those related to ATP and NADPH, sucrose (Suc), and malate (Outlaw, 1989; Reckmann et al., 1990; Lawson et al., 2003; Lawson et al., 2014). Some studies support that the apoplastic Suc, where occurs in some cell walls of GCs from mesophyll cells as an osmoticum, may be responsible for the stomatal opening (Lawson et al., 2003; Kang et al., 2007). An early starch-sugar hypothesis suggested sugars, such as Suc, which is the end product of photosynthesis, may be derived from starch degradation in mesophyll cells and may play an important role in linking mesophyll photosynthesis to GC function (Pallas, 1964; Tallman and Zeiger, 1988; Ni, 2012). However, this hypothesis is still not well tested. With the ambiguous role of sugars in stomatal regulation, the role of Suc as a major osmoticum driving stomatal movement has been debated. However, Suc may still play a critical role in interrelating mesophyll and stomatal behavior, possibly via apoplasts. Thus, Suc role is implicated in a feedback-inhibition mechanism with an expression of hexokinase (HXK) in GCs when the Suc production rate exceeds the efflux rate at which Suc is loaded into the phloem (Kelly et al., 2013) under elevated CO_2_ conditions (Cheng et al., 1998; Long et al., 2004; Ainsworth and Rogers, 2007). A HXK-induced expression of ABA-related genes leads to a decrease in the influx of apoplastic sugar entering the GCs from the mesophyll, which may coordinate photosynthesis with transpiration by coupling with a stomatal closure (Kang et al., 2007; Kelly et al., 2013). It highlights the pivotal role of HXK. Moreover, a limitation to a carbon sink or transportation of sugar from shoot to root via the phloem leads to the accumulation of sugar in shoots and/or leaves and results in stomatal closure. This strengthens the hypothesis of sugar-driven stomatal movement (Domec and Pruyn, 2008; Silber et al., 2013).

#### Gene Involvement

The negative regulation of elevated CO_2_-induced stomatal closure may be closely linked to an impaired Ca^2+^ priming sensor, a HT1 protein kinase, and an RHC1 MATE-type transporter in *Arabidopsis* plants (Hashimoto et al., 2006; Young et al., 2006; Negi et al., 2014; Tian et al., 2015) (**Table 1**). However, the underlying mechanism concerning the precise signal transduction molecular pathways that regulate the stomatal closure upstream still remains elusive. This needs to be explored further, particularly for different genetic types, species, and even PFTs. A repression of the ABC transporter AtABCB14 may play a considerable role in stomatal closure in response to elevated CO_2_ levels (Lee et al., 2003, 2008; Laanemets et al., 2013). This SLAC1 may also be involved in stomatal closure induced by elevated CO_2_ levels (Negi et al., 2008; Laanemets et al., 2013). A recent report indicated that SLAC1 perception of CO_2_ signals may be located in a transmembrane region by an ABA-independent pathway (Yamamoto et al., 2016). Phosphorylation of KAT1 on the C-terminal region, which is expressed primarily in GCs in *A. thaliana* plants, might modulate the activity of K^+^ channels involved in the signal transduction cascade (Sato et al., 2009), which might be negatively regulated by nitric oxide (NO)—an active signaling molecule in plants (Gayatri et al., 2013). ABA may trigger its generation (Neill et al., 2008; Shi et al., 2014; Xia et al., 2015) through the modulation of vitamin B_6_ homeostasis (Xia et al., 2014). Furthermore, because blue light photoreception may also be involved in light–CO_2_ interactions in GCs, the changes in zeaxanthin levels may correspond to changes in the CO_2_ level, which are linked to the pH sensitivity of the relevant enzymes (Zeiger and Zhu, 1998; Zhu et al., 1998). A recent report indicated that NADPH oxidases and respiratory burst oxidase homologs (RBOHs) were closely associated with the network of reactive oxygen species (ROS) production, which may regulate the stomatal aperture (Baxter et al., 2014).

#### Mesophyll-Derived Signal (MDS)

Malate generated in GCs, through the metabolite of triose phosphate (triose-P) from the Calvin–Benson–Basshan (CBB) cycle, may directly involve stomatal aperture regulation as an osmoticum and as a sink for the end products of GC electron transport involving phosphoenolpyruvate carboxylase (PEPC; Cousins et al., 2007; Lawson et al., 2014). A component of malate may also originate from mesophyll cells because when the tricarboxylic acid (TCA) cycle function has been limited, e.g., by the inhibition of fumarase (Nunes-Nesi et al., 2007), there is a decline in GC malate, as it is one of the metabolites derived from the TCA cycle in mesophyll cells (Fernie and Martinoia, 2009; Araújo et al., 2011). It might confirm that malate could be the mesophyll-derived signal (MDS) linking stomatal behavior. A negative correlation between the fumarate level in mesophyll and *g*_s_ indicated that fumarate, as an MDS, may also be involved in stomatal closure, although its influence seems to be less than that of malate (Araújo et al., 2011; Medeiros et al., 2015). Moreover, high CO_2_ concentration-induced stomatal closure may be attributable to an increase in the concentrations of malate produced in the mesophyll stimulating anion efflux through, for example, the R-type channel (ALMT). This is a malate-sensitive anion channel operating as a CO_2_ sensor in GCs and is linked to mesophyll photosynthesis (Hedrich and Marten, 1993; Sasaki et al., 2010; De Angeli et al., 2013; Lawson et al., 2014; Medeiros et al., 2015).

However, whether mesophyll and/or guard cell photosynthesis is involved in the GC response to CO_2_ concentrations remains controversial (von Caemmerer et al., 2004; Messinger et al., 2006; Lawson et al., 2014). Early reports show that a specific blue light response involving H^+^-ATPase activation is independent of *A*, whereas the red light response may be associated with *A*, which might be induced by the intercellular CO_2_ concentration (*C*_i_) reduction resulting from the mesophyll consumption of CO_2_ (Roelfsema et al., 2002; Messinger et al., 2006). A recent study showed that *Arabidopsis* plants with an overexpression of plasma membrane H^+^-ATPase under the control of a guard cell-specific promoter may facilitate the coordinative capacity between stomatal opening, *A*, and growth rate (Wang et al., 2014). Thus, the role of photosynthesis in regulating GC movement in response to elevated CO_2_ remains elusive (Roelfsema et al., 2002; Ainsworth and Rogers, 2007; Engineer et al., 2014). The relative role of photosynthesis in guard cells and the nearby related cells, such as mesophyll cells, in response to elevated CO_2_ may require further testing.

There is no clear evidence for or against the existence of MDS and the involved signals. Some potential signals, such as chloroplastic ATP, zeaxanthin, NADPH, RuBP, and stomatin, have been suggested (cf. Lawson et al., 2014). Support for the role of MDS has been found in some excellent experiments, such as those by epidermal peels vs. the intact leaves methods. These experiments yield strong evidence that MDS might occur (e.g., Roelfsema et al., 2002; Mott et al., 2008; Fujita et al., 2013). Some reports indicate the MDS may exist in modern seed plants rather than in ferns and lycophytes (e.g., McAdam and Brodribb, 2012). Additionally, MDS may need certain transduction medium conditions, such as a vapor phase (Sibbernsen and Mott, 2010) or aqueous phase (Fujita et al., 2013). With increasing evidence that *C*_i_ may play only a trivial role (von Caemmerer et al., 2004; Hanson et al., 2013), the biological activities closely related to MDS often refer to electron transport, the redox state, metabolites in the transpiration stream, vapor phase ion, and electrical signals (Lawson et al., 2014). A report indicated that stomatal opening linked to apoplast transfer from mesophyll signals is dependent on photosynthesis at lower levels of CO_2_ (Fujita et al., 2013). Moreover, the stomatal closure is relatively independent of photosynthesis at elevated CO_2,_ i.e., without ATP involvement in mesophyll photosynthesis (Roelfsema et al., 2002; Fujita et al., 2013). The S-type anion channels activated at elevated CO_2_ may contribute to stomatal closure (Roelfsema et al., 2002; Fujita et al., 2013). A study using chlorophyll fluorescence imaging showed spatiotemporal decoupling of stomata and mesophyll in response to the cutting of leaf veins, which weakens further support for the appearance of MDS (Hanson et al., 2013).

#### Integrated Signaling Processes

The changes in stomatal development and its aperture induced by elevated CO_2_ and involving mesophyll conductance (*g*_m_; Mizokami et al., 2015; Youshi and Santrucek, 2015) might be mediated by ABA levels (Giday et al., 2014; Youshi and Santrucek, 2015). In a recent study, genetic analysis using mutants in the ABA signaling pathway on GC-specific transcriptional memory for the related genes indicated that SnRK2.6 is more important for overall stomatal control. The SnRK2.2 and SnRK2.3 are more important for implementing GC stress memory in the subsequent dehydration response (Virlouvet and Fromm, 2015). However, the involvement of SnRK2.2 and SnRK2.3 in elevated CO_2_ regulation on the stomatal response and feedback remains largely unclear. The long-distance signaling cascades (Lake et al., 2002), e.g., from mature leaves to immature leaves, may also contribute to the GC behavior response to CO_2_ levels. ABA, ethylene, salicylic acid (SA), jasmonic acid (JA), NO, some peptides, and sugar levels might be involved in the integrated signaling processes’ response to environmental changes (e.g., Neill et al., 2008; Poór et al., 2011; Silber et al., 2013; Xia et al., 2014, 2015; Grienenberger and Fletcher, 2015; Medeiros et al., 2015).

## Interactions with Other Factors

### Elevated CO_2_ with Drought

Soil water deficit and high VPD often reduce the stomatal opening, depending on the species (Warren, 2008; Perez-Martin et al., 2009; Peak and Mott, 2011). Generally, water status has a stronger impact on *g*_s_ than changes in CO_2_ concentration. A relatively small effect of elevated CO_2_ on *g*_s_ generally appears as water deficit stress occurs, possibly because the drought-induced reduction dramatically outweighs the reduction caused by elevated CO_2_ (Morgan et al., 2004; Leakey et al., 2006b). Flexas et al. (2004) indicated that decreases in *g*_s_ and *g*_m_, but not biochemical activities, may limit the photosynthetic capacity in drought-stressed leaves, depending on the species (Bota et al., 2004; Flexas et al., 2014). Even for drought-severely stressed plants, the biochemical limitation can be negligible (Galmés et al., 2007). A non-stomatal limitation appears only when *g*_s_ is below 250 mmol m^-2^s^-1^ in grass plants grown in drought conditions (Xu et al., 2009a). In tall fescue (*Festuca arundinacea*) plants exposed to elevated CO_2_, an increased *A* with a low *g*_s_ but high Rubisco activity during both drought and rewatering may also indicate the alleviation of metabolic limitations caused by drought damages rather than stomatal limitations imposed by elevated CO_2_ (Chen et al., 2015). CO_2_ enrichment may relieve non-stomatal limitations by protecting the photosynthetic apparatus during severe drought (Xu et al., 2014). However, a recent report showed that *Ramonda nathaliae* plants with smaller stomata have higher resistance to drought than *R. serbica*, which have larger stomata (Rakić et al., 2015). This highlights the role of the stomatal size.

**FIGURE 3:**
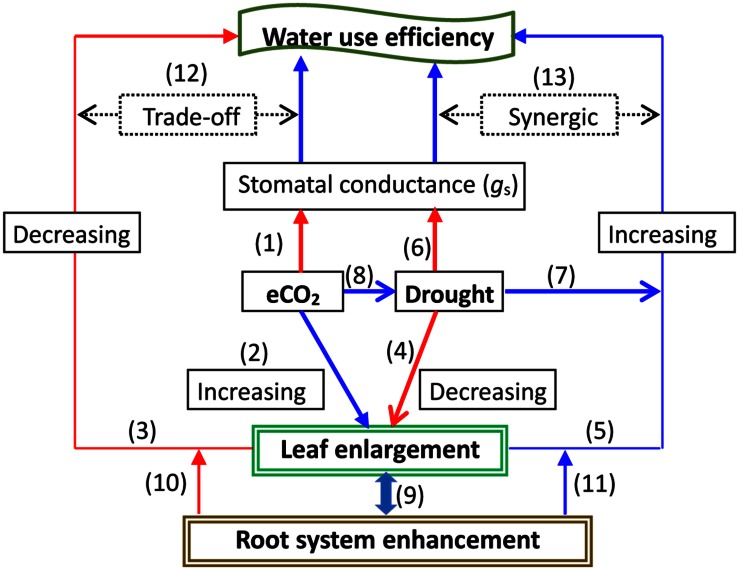
**A representation of the response to elevated CO_2_ (eCO_2_) with drought on water use efficiency (*WUE*) under regulations by balancing stomatal conductance (*g*_s_) and leaf growth.** Elevated CO_2_ may lead to an acclimated reduction in *g*_s_, which involves signaling sensing and transduction, biophysical and biochemical processes, and gene expression (1); meanwhile, eCO_2_ could promote leaf enlargement (2), possibly increasing transpiration (*E*) of the total leaf subsequently reducing *WUE* (3). A severe drought stress may shrink leaf growth (4), consequently decreasing *E* and finally increasing *WUE* (5); *g*_s_ can directly be reduced by drought (6). However, a moderate drought may directly enhance *WUE* by some adaptive responses such as a relative increase in the root systems (7), which can be further improved by elevated CO_2_ (8). Root systems may be enhanced by eCO_2_, particularly under drought through alterations in carbon allocation between above- and belowground parts (9), which may lead to either decreased *WUE* at eCO_2_ (10), or increased *WUE* under drought conditions (11). Consequently, this trade-off interaction (12) or synergic increase (13) may occur with leaf growth and *g*_s_ changes at eCO_2_ under drought, ultimately affecting *WUE*.

Elevated CO_2_ may improve plant water status by reducing *g*_s_ and thereby raising *WUE*, ameliorating the adverse effects of stressful factors on plant growth and physiological processes (Ainsworth and Rogers, 2007; Xu et al., 2013, 2014). A decrease in soil water availability under elevated CO_2_ may be closely linked to an increase in leaf area, which offsets a decline in *g*_s_ and promotes plant growth (Manea and Leishman, 2015). Studies have clearly shown that water status mediates rising CO_2_ effectiveness through the coupling of processes between gas exchange and leaf enlargement. Nevertheless, the pros and cons of acclimation to changes in water conditions may coexist in response to elevated CO_2_. Leaf area enlargement, i.e., canopy enhancement induced by CO_2,_ may exaggerate water use, whereas decreased *g*_s_ would promote *WUE* (e.g., Woodward, 1990; Ward et al., 2013; Manea and Leishman, 2015), depending on canopy density and its homogeneity (Bernacchi and VanLoocke, 2015). However, an intrinsic *WUE* decline might appear during severe drought in some relict species plants exposed to elevated CO_2_ (Linares et al., 2009). Thus, future research is necessary to focus on the linkage among leaf area, *g*_s_, and both *WUE*_i_ and total plant biomass water use efficiency (*WUE*_t_) under climatic change. Furthermore, some results indicated that although *WUE*_t_ and *WUE*_i_ showed a similar response to elevated CO_2_, the former seemed to have a higher level of sensitivity, implying that *WUE*_t_ may be a better indicator than *WUE*_i_ of the response to climate change (Duan et al., 2014). *WUE* and the root: shoot biomass ratio increased significantly with decreased precipitation but decreased with elevated CO_2_ levels (Li et al., 2014). Thus, besides the regulation of leaf growth, root development may also involve stomatal movement behavior and *WUE* changes under climatic change. The possible primary stomatal closure induced by elevated CO_2_ may be offset by positive indirect effects on *g*_s_, possibly caused by root system promotion and hydraulic capacity under rising CO_2_ conditions (Uddling et al., 2009). Forest canopy evapotranspiration can be reduced under high CO_2_ concentration levels (Medlyn et al., 2001), possibly due to leaf *g*_s_ slowdown. Thus, water loss is diminished. However, a lower response to elevated CO_2_ in the canopy evapotranspiration rate relative to leaf *g*_s_ was found in a rice field (Shimono et al., 2013). Nevertheless, the canopy carbon fixation and its association with *g*_s_ at the leaf and canopy scales during climatic change remains to be tested. A succinct description on the trade-off between *g*_s_, leaf enlargement, and *WUE* under elevated CO_2_ and drought conditions is summarized in **Figure 3.**

Moreover, most studies have confirmed that elevated CO_2_ may improve the water status of drought-stressed plants by reducing *g*_s_ (e.g., Brodribb et al., 2009; Katul et al., 2010; Chen et al., 2015; Easlon et al., 2015), but these findings were species-dependent (Beerling et al., 1996; Bernacchi et al., 2007; Liu et al., 2016). However, this case may not occur under severe or extreme drought conditions, possibly due to the depression of stomatal regulatory ability (Xu and Zhou, 2008). Furthermore, plant size and root distribution may override the expected direct physiological effects of elevated CO_2_ (Duursma et al., 2011; Liu et al., 2016).

### Elevated CO_2_ with Salinity

Generally, stomata may exert a similar response to salt stress relative to drought (Clough and Sim, 1989; Wang et al., 2003; Flexas et al., 2004; Chaves et al., 2009). Stomatal conductance often decreases remarkably with increased salinity and/or aridity, such as leaf to air VPD, depending on the species and its habits (e.g., Clough and Sim, 1989; Chaves et al., 2009; Ashraf and Harris, 2013; Nguyen et al., 2015; Sanoubar et al., 2016). Enhanced salt stress and elevated CO_2_ concentrations are projected to co-occur in the future (Chaves et al., 2009; Pérez-López et al., 2009; Hoque et al., 2016). Generally, stomatal conductance was decreased by severe salt stress and elevated CO_2_ alone or in combination (Pérez-López et al., 2012; Nguyen et al., 2015; Stavridou et al., 2016). For example, as barley (*Hordeum vulgare*) plants are grown in high salinity soil, the rate of CO_2_ diffusion to the carboxylating site and photochemical electron sink capacity increased under elevated CO_2_ conditions, despite stomatal and internal conductance being decreased (Pérez-López et al., 2012). Similar to the severe desiccation effect, high salinity stress may lead to oxidative damage in plant tissue (Shalata et al., 2001; Sanoubar et al., 2016). However, elevated CO_2_ may alleviate the oxidative stress-induced by salinity with lower ROS level and a higher *A*, thus improving plant growth under high salinity conditions (Nicolas et al., 1993; Pérez-López et al., 2009). Studies have indicated that the rising-CO_2_ protection from salt-inhibited plants alleviates the metabolic limitations rather than the stomatal limitations. Moreover, although there was a *g*_s_ decrease of 1–2 factors by high soil salinity in wetland grass *Phragmites australis* plants, the salinity effect hardly occurred with the combination of elevated CO_2_ and temperature (plus 310 μmolmol^-1^ CO_2_, and plus 5°C relative to ambient variables; Eller et al., 2014). The non-species expansion into saline areas may be promoted because the salinity-caused non-stomatal limitations (i.e., carboxylation rates of Rubisco or electron transport rates) may be mitigated under the elevated climatic conditions (Eller et al., 2014). However, the alleviated effect of elevated CO_2_ on severe salt stress strongly depends on species and cultivars/ecotypes (Eller et al., 2014; Geissler et al., 2015). Nevertheless, the responses of stomatal characteristics to the combination of elevated CO_2_ on salt stress are scarcely reported and need to be explored further.

### Elevated CO_2_ with High Temperatures

The combined effects of elevated CO_2_ and high temperatures have also been reported in some studies. While there are exceptional cases (e.g., Bernacchi et al., 2007), elevated CO_2_ decreases *g*_s_, thus increasing leaf temperature because lower transpiration releases less heat (Kim et al., 2006; Negi et al., 2014; Šigut et al., 2015). As a consequence, elevated CO_2_ with high temperatures may play an antagonistic role by exaggerating heat damage partly due to decreased *g*_s_ (Warren et al., 2011). However, an elevated CO_2_-induced 13–30% decline in *g*_s_ induced a 2°C increase in leaf temperature, leading to a 2.9–6.0°C increase in the temperature optima for the light-saturated rate of CO_2_ assimilation (*A*_max_). Thus, this would enhance heat stress tolerance in beech and spruce saplings (Šigut et al., 2015). The increased adaptation to heat stress may be due to reduced photorespiration and the limitation of photosynthesis by RuBP regeneration under elevated CO_2_ (Šigut et al., 2015). A recent report also confirmed the heat-tolerance enhancement due to elevated CO_2_ for coffee crops (Rodrigues et al., 2016). Thus, the negative effect of elevated CO_2_ on heat stress due to reduced *g*_s_ was not confirmed. In contrast, a beneficial adaptation may occur. Yet, this may depend on the species and the range of temperature variation.

### Elevated CO_2_ with Nutrition Status and Air Pollution

Based on a recent report (Easlon et al., 2015), better plant growth and photosynthesis in the low *g*_s_ in *A. thaliana* lines under N-limitation, rather than sufficient N supply under elevated CO_2_, may imply an adaptive coupling between lowered *g*_s_ and improved N utilization. Increased conservative N investment in photosynthetic biochemistry in order to acclimate to CO_2_ fertilization highlights a positively synergistic relationship between stomatal regulation and nutrition status. However, a lower *g*_s_ in elevated CO_2_ concentrations but a higher *g*_s_ with an abundant N supply have been found in *Liquidambar styraciflua* plants (Ward et al., 2013), suggesting that these factors may play opposite roles in the *g*_s_ response. A recent study has indicated that improved phosphorus (P) nutrition can enhance drought tolerance in the field pea due to the CO_2_-induced decrease in *g*_s_ and the promotion of root systems (Jin et al., 2015). A general decline in *g*_s_ by elevated CO_2_ and ozone (O_3_) alone or their combination has been extensively reported, suggesting that rising CO_2_ may alleviate the injury caused by high O_3_ pollution decreasing *g*_s_ (Kellomäki and Wang, 1997; Mansfield, 1998; Warren et al., 2006; Hoshika et al., 2015). However, some species, such as aspen (*Populus tremuloides* Michx.) and birch (*Betula papyrifera* Marsh.), have a high *g*_s_ under both high CO_2_ and high O_3_ concentrations (Uddling et al., 2009). This indicates that the interactive effects between elevated CO_2_ and O_3_ on stomatal behavior may depend on species, plant/leaf ages, and treatment regimens, such as time and sites (Uddling et al., 2009; Hoshika et al., 2015; Matyssek et al., 2015). Thus, it again highlights the complex/specific response.

### Elevated CO_2_ with Biotic Factors

The stomatal response to elevated CO_2_ with biotic factors has received much attention (e.g., Casteel et al., 2012; Zavala et al., 2013). For instance, a greater *g*_s_ reduction in cabbage with decreased aphid (one of the most destructive insect pests in crops) colonization rates and total plant volatile emissions, such as terpene emissions, occurred when plants were exposed to elevated CO_2_ over the long-term (6–10 weeks) rather than the short-term (2 weeks; Klaiber et al., 2013). This indicates that, as hosts, plants may acclimatize to future increases in elevated CO_2_ by modifying stomatal behavior. Under elevated CO_2_, a decrease in micronutrients, such as calcium, magnesium, or phosphorus, due to the g_s_ reduction may lead to poor aphid performance (*Myzus persicae*; Dáder et al., 2016). Furthermore, a recent report (Sun et al., 2015) showed that aphid infestation may synergistically promote the effects of elevated CO_2_ on stomatal closure, possibly by triggering the ABA signaling pathway. Therefore, the water status of the host plants of *Medicago truncatula* was improved, ultimately enhancing feeding efficiency and abundance of aphid (Zavala et al., 2013; Sun et al., 2015). Taken together, plant–insect interactions might be modified by stomatal closure under high levels of CO_2_. The metabolism and emission of plant biogenic volatile organic compounds may also be involved (Klaiber et al., 2013; Zavala et al., 2013). It is suggested that an enhanced accumulation of JA and SA may also be involved in signal transduction in relation to stomatal movement as plants are subjected to CO_2_ enrichment and herbivore attack. This highlights an important role in stomatal regulation to cope with a combination of climate change and biotic factors (Poór et al., 2011; Casteel et al., 2012; Zavala et al., 2013; Sun et al., 2015). Thus, the herbivore’s adaptive capacity to its host might be promoted when exposed to elevated CO_2_, at least partly through stomatal regulation.

## Conclusion and Perspectives

Under high CO_2_ conditions, both stomatal conductance and its density generally decreased with a few exceptions. The decline in SD may be the result of a long-term genetic variation or short-term structural plasticity under elevated CO_2_. Elevated CO_2_ may induce the excessive depolarization of guard cells to cause stomatal closure when mesophyll-driven signals, such as malate, ATP, zeaxanthin, and NADPH, may be involved in stomatal movement. Their photosynthesis in both guard cells and mesophyll cells and their link to the stomatal response in elevated CO_2_ conditions may play an important role. However, challenges remain in elucidating the underlying mechanism. The differences and linkage in stomatal responses to elevated CO_2_ levels across the molecular, cellular, biochemical, eco-physiological, canopy, and vegetation levels (Zhu et al., 2012; Peñuelas et al., 2013; Shimono et al., 2013; Armstrong et al., 2016) should raise concerns about ecological and climatic management.

Several crucial aspects of research into the stomatal response may need to be strengthened in the future. (1) The underlying mechanism of responses to CO_2_ enrichment for key biological processes, including stomatal behavior; the critical metabolic bioprocesses, such as hormone-involved regulation; and relevant biochemical signal cascades must be further elucidated. (2) The diverse responses from different species and PFTs to elevated CO_2_ or its combination with other abiotic and biotic factors must be compared and clarified. (3) Various spatial–temporal scales from the molecular, biochemical, physiological, individual, and canopy to vegetation levels must be integrated. Instantaneous to annual or longer time-scales (e.g., Zhu et al., 2012; Shimono et al., 2013; Armstrong et al., 2016) must also be integrated. We should elucidate the underlying mechanism of the stomatal responses associated with key biological processes across the multiple scales under different climatic factors, including elevated CO_2_, warming, drought, and air pollution. (4) We need to investigate whether improving stomatal response to elevated CO_2_ by manipulating guard cell performance may yield a better balance between CO_2_ uptake and water loss through transpiration to enhance photosynthetic capacity with high *WUE* (e.g., Engineer et al., 2014; Lawson and Blatt, 2014; Grienenberger and Fletcher, 2015). Enhanced expression of some related genes, such as *patrol1*, may drastically increase both *g*_s_ and plant growth under higher CO_2_ levels (Hashimoto-Sugimoto et al., 2013). This task needs to be implemented urgently. Finally, understanding how to improve or combine earth system models (ESMs), general circulation models (GCMs), and land surface models (LSMs) may help to correctly interpret the *g*_s_ response to climate change (Sato et al., 2015). The integration issue should be solved urgently to precisely assess the response and feedback of terrestrial ecosystem to global change.

## Author Contributions

YJ and BJ are co-first authors, ZX and GZ designed the study, ZX, YJ, and BJ collected and analyzed the data, all authors wrote and reviewed the manuscript.

## Conflict of Interest Statement

The authors declare that the research was conducted in the absence of any commercial or financial relationships that could be construed as a potential conflict of interest.
